# Stakeholder perspectives for optimization of tuberculosis contact investigation in a high-burden setting

**DOI:** 10.1371/journal.pone.0183749

**Published:** 2017-09-08

**Authors:** Diana Marangu, Hannah Mwaniki, Salome Nduku, Elizabeth Maleche-Obimbo, Walter Jaoko, Joseph Babigumira, Grace John-Stewart, Deepa Rao

**Affiliations:** 1 Department of Paediatrics and Child Health, University of Nairobi, Nairobi, Kenya; 2 Institute of Tropical and Infectious Diseases, University of Nairobi, Nairobi, Kenya; 3 Population Studies and Research Institute, University of Nairobi, Nairobi, Kenya; 4 Semantics Africa Limited, Nairobi, Kenya; 5 Department of Medical Microbiology, University of Nairobi, Nairobi, Kenya; 6 Department of Global Health, University of Washington, Seattle, Washington, United States of America; 7 Department of Allergy and Infectious Disease, University of Washington, Seattle, Washington, United States of America; 8 Department of Epidemiology, University of Washington, Seattle, Washington, United States of America; 9 Department of Paediatrics, University of Washington, Seattle, Washington, United States of America; 10 Department of Psychiatry and Behavioral Sciences, University of Washington, Seattle, Washington, United States of America; McGill University, CANADA

## Abstract

**Introduction:**

Optimal tuberculosis contact investigation impacts TB prevention, timely case finding and linkage to care, however data on routine implementation in high burden contexts is limited.

**Materials and methods:**

In a multi-method qualitative study based on individual interviews with TB patients, facility observations and focus group discussions with health workers (HWs) in 13 public health facilities, and key informant interviews with governmental and non-governmental experts, we describe TB contact investigation in the context of an urban setting in Kenya and identify opportunities for optimization.

**Results:**

Invitation of TB patients to bring close contacts by HWs was key for all patient decisions that led to contact screening in addition to patients’ understanding of TB transmission and desire to avoid contacts suffering from TB. Sub-optimal HW enquiry of TB patients and contacts presenting at the facility were missed opportunities which stemmed from lack of standardized operational procedures, documentation tools and HW training. Stakeholders proposed provision of fast tracked and holistic health packages for contacts seeking TB screening, and sustainable government led funding for the requisite infrastructure and workforce.

**Conclusion:**

TB contact invitation by HWs leading to contact screening occurs in this context. Stakeholder perspectives inform the design of an operational framework for optimized delivery.

## Introduction

In 2015, it was estimated that 41% of the 10.4 million people with active tuberculosis (TB) worldwide were undiagnosed or unreported [1]. In a meta-analysis of 71 studies including 878,724 participants in low and middle income countries (LMICs), pooled prevalence of TB among persons who were exposed to TB was 3.1%, and in 76 studies pooled prevalence of latent TB infection was 51.5% among 60,557 contacts screened [2]. The World Health Organization (WHO) considers contact investigation (CI) an efficient approach to intensified TB case finding and it is one of 10 indicators of the End TB Strategy, with a recommended target level of ≥ 90% by 2025 [1, 3]. The TB-CI strategy identifies asymptomatic individuals, including children and HIV infected individuals, who are at high risk for TB disease [4–9]. TB-CI is a gateway to isoniazid preventive therapy (IPT) delivery and part of TB care. Although CI has been shown to be feasible in some LMICs [10], data on routine TB-CI implementation in high TB burden contexts is scarce.

The WHO advises that TB-CI may be of value in high HIV/multidrug resistant (MDR) TB contexts as these are known to reduce TB treatment success rates [7, 11]. Gaps in specific policy, guidelines, tools and financial resources for CI scale-up have been recognized in these settings. However, the implementation of routine TB-CI has not been formally assessed [12]. We sought to examine TB-CI by characterizing experiences of index TB patients seeking care in Nairobi, a densely populated city in a high burden HIV, TB and MDR-TB LMIC. In addition, we sought to identify and describe health system facilitators, barriers and opportunities for optimization from the perspectives of health workers (HWs) and experts involved in TB-CI related care provision.

## Materials and methods

Following ethical approval from the University of Nairobi-Kenyatta National Hospital Ethics and Research Committee and the University of Washington Institutional Review Board, we conducted this multi-method qualitative study between April 2015 and July 2016.

### Research team and reflexivity

The team included senior researchers with extensive experience in the fields of qualitative research, TB-HIV and health economics. The first author, a Kenyan pediatrician with formal training and experience in qualitative research made facility observations and conducted individual patient interviews, key informant interviews (KIIs) and focus group discussions (FGDs) with study participants without establishing a relationship prior to the start of the study. The second author, a Kenyan population studies researcher and anthropologist with extensive qualitative research experience transcribed all the interviews. These two multi-lingual researchers independently coded the transcripts and resolved differences in the analysis by consensus. The third author, a Swahili/English Translator and Communicator with over 10 years of experience in translation and localization, independently reviewed the TB patient interviews to ensure recommendations proposed accurately reflected patient needs.

### Participant selection

**Fig 1:**
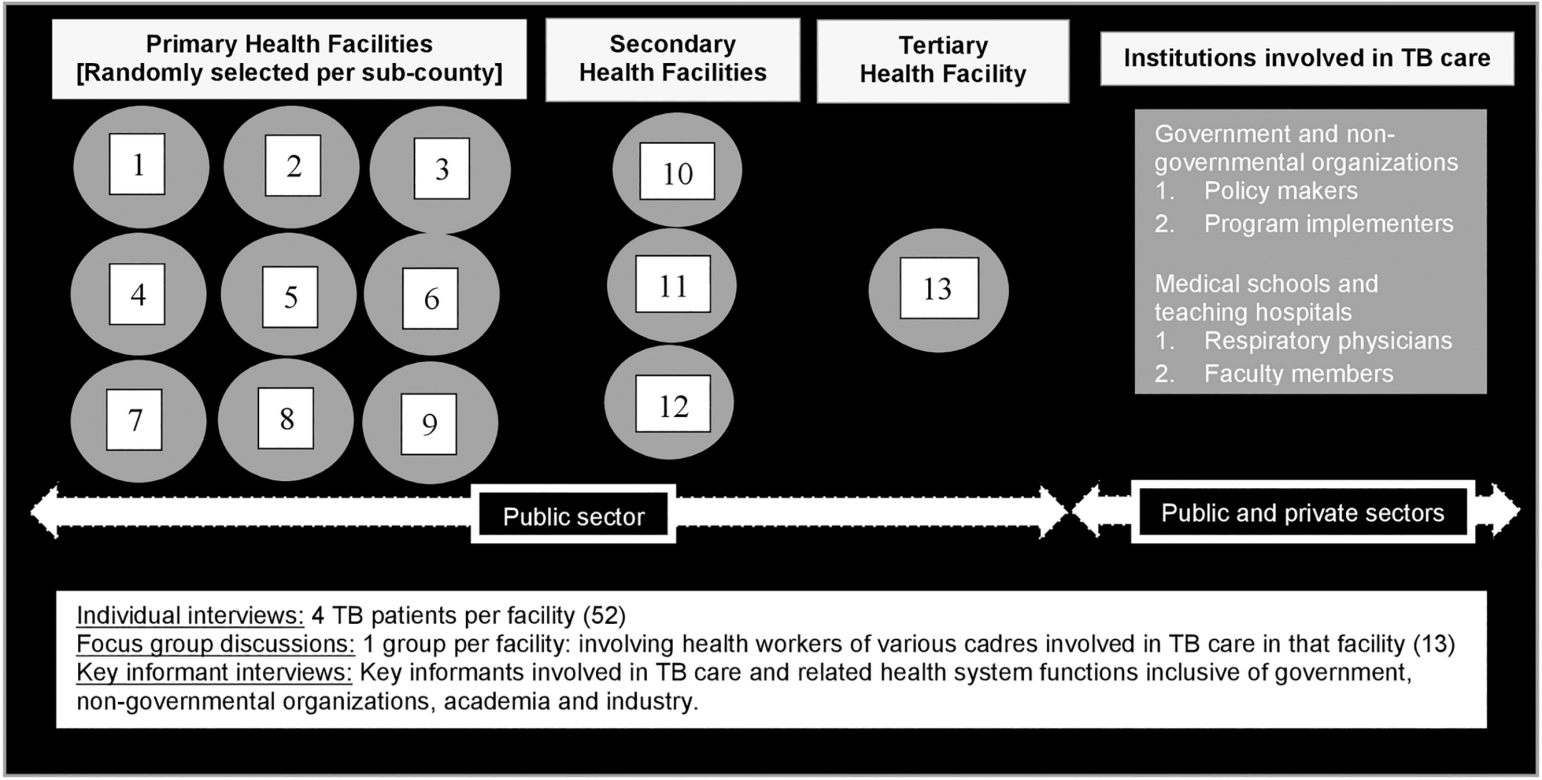
Schema of sampling frame of stakeholders involved in TB care in Nairobi County, Kenya.

Participants comprised of three groups of stakeholders: TB patients, HWs, and experts involved in various areas of TB care in Kenya. As described in the Kenya Health Policy 2014–2030, the health delivery system is progressively transforming into four tiers of care: community, primary care, secondary referral care, and tertiary referral care. Level II (dispensaries) and level III (health centres) facilities are to provide primary care, whereas level IV (primary care hospitals) and level V (secondary care hospitals) facilities will provide secondary referral care [13]. We selected study participants from 13 TB clinics in public health facilities in Nairobi, Kenya, stratified by the level of health care provided as shown in Fig 1. These included all tertiary and secondary public referral facilities in Nairobi County offering TB services. We also randomly sampled one facility from each of the nine sub-counties among 27 public primary health facilities listed in the 2014 master-list of health facilities [14].

#### TB patients

Index TB patients were selected to gain individual perspectives in decision making for TB screening, and were eligible if they had clinician-diagnosed pulmonary TB, were aged ≥18 years and attending the selected TB clinics. On two separate visits at each selected TB clinic, the researcher consecutively selected the first male and female patients from the TB patient register at the time of the visit. Where gender balance was not possible at the visit, a second participant of the same gender was conveniently selected. Therefore four patients from each of the 13 health facilities were selected with the goal of having a balanced sex distribution where possible.

#### Health workers

We selected HW provider teams responsible for TB-CI at each of these selected clinics to gain their ‘on the ground’ perspective. These teams comprised of various cadres involved in TB care including nurses, clinical officers, community health workers (CHWs), HIV counselors, social workers and laboratory technicians. The teams’ working relationship was leveraged to provide a safe environment to generate and gain valuable insights during the focus group discussions.

#### Key informants

Key informants who were experts involved in diverse aspects of TB-CI including policy makers, program implementers, academia, and industry, were purposefully selected to gain an in depth understanding of the health system in relation to TB-CI.

The lead researcher invited these stakeholders to participate in the study, sought informed written consent from each participant and conducted face-to-face interviews and FGDs during hours of facility or institution operation.

### Data collection process

**Fig 2:**
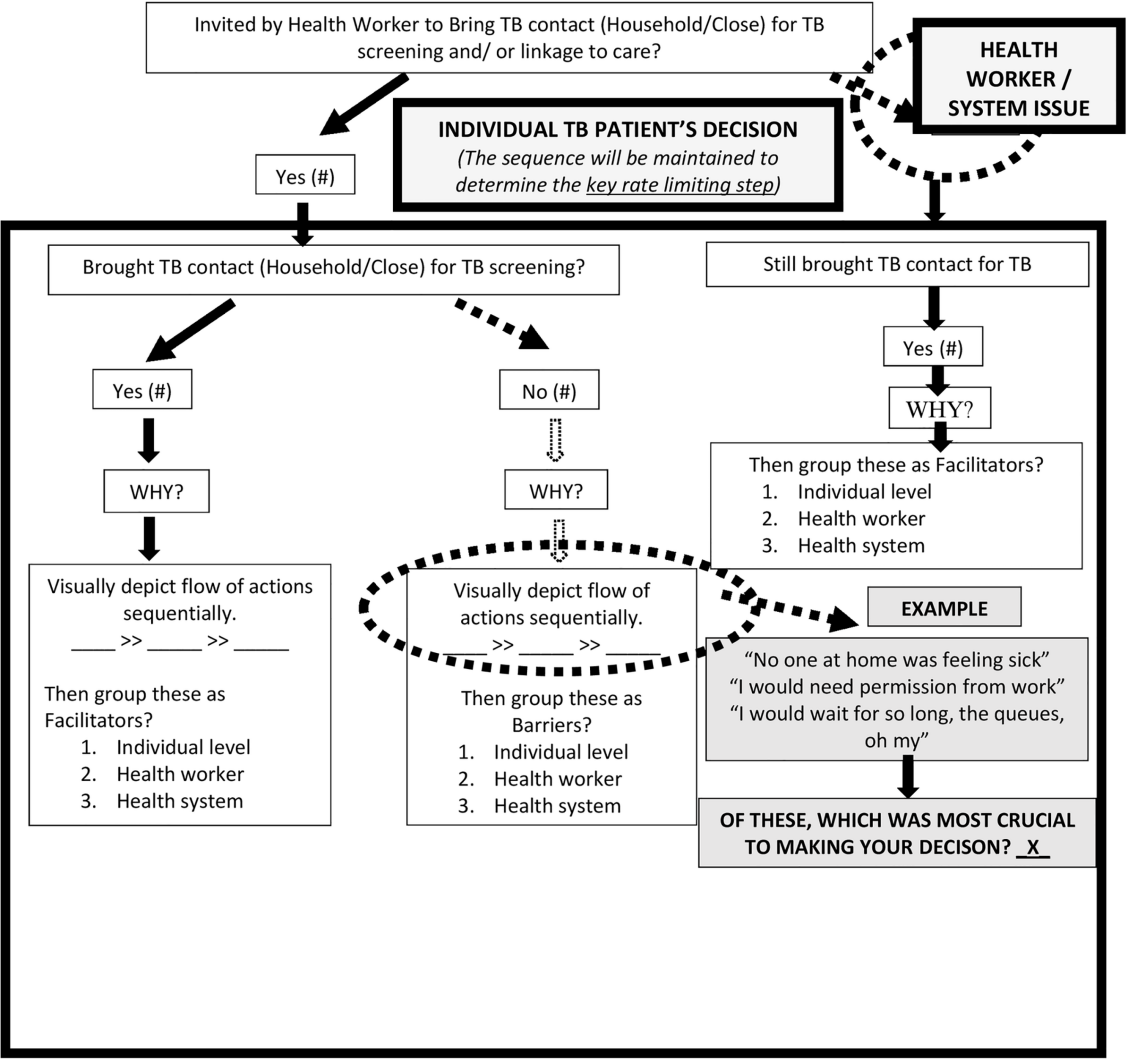
Hypothetical decision model: Index TB patients’ decisions on contact investigation.

Prior to study commencement, the research team created a hypothetical TB-CI decision model for index TB patients with plausible decision arms based on existing literature and prior clinical experience. (Fig 2) The lead researcher used this hypothetical model to guide open ended interviews with individual TB patients. After obtaining informed consent, the researcher established rapport, enquired about pertinent sociodemographic characteristics such as a) age, b) education level, c) occupation, d) duration since TB diagnosis, e) living dynamics with regard to household and close contacts, f) where TB patients spent most of their time including travel, g) processes that took place at the health facility, h) the health education given by the HW and i) close contacts and the general TB care provided. Thereafter we enquired about facilitators, barriers and opportunities for TB contact screening optimization at each decision node. Our facility observation flow mapping guide and focus group discussion/interview guide are provided. (S1 and S2 Tables) To guide FGDs and documentation of facility observations including mapping flow of TB-CI related activities, we used the WHO’s six building blocks framework. These building blocks include: 1) health service delivery, 2) health workforce, 3) health information systems, 4) access to essential medical products, vaccines and technologies, 5) health systems financing and 6) leadership and governance. Since health systems are highly context-specific, this framework enables a health system to be analyzed in its totality, utilizing a unified approach that allows for identification of strengths and gaps [15]. In addition to the insights gained from HWs during the FGDs regarding general and TB-CI care at their facilities, we also provided scenarios to gauge the spectrum of TB-CI actions specific to contacts who were children or who were HIV-infected. During individual interviews, FGDs and KIIS, no one else was present during data collection besides the participants and lead researcher. The researcher audio recorded the interviews; took field notes and noted when data were saturated. Interviews were transcribed verbatim by the second author. We did not repeat interviews or return transcripts to participants for comments or correction. To maintain confidentiality, we did not audio record participant names during interviews. We stored data in password protected files with access restricted to researchers, and destroyed audio-recorded files six months after transcription as per our protocol.

### Analyses

We were able to validate our data by cross verifying multiple sources (health facilities, TB patients, HWs and key informants) and utilizing different qualitative methodologies (observations, interviews and focus group discussions). Data analysis was at three levels: patient, HW and health system. At the patient level analysis, we assessed the TB-CI decision model based on two decision levels: Was the TB patient invited by a HW to bring his/her close contacts for TB screening? Were any of the close contacts screened for TB? At each decision node we identified facilitators, barriers and opportunities for TB-CI optimization and analyzed themes. We also analyzed data from facility observations, HW FGDs and stakeholder KIIs, and synthesized themes using WHO’s six health system building blocks [15]. To ensure data validity, the lead researcher and second author independently analyzed the data and settled differences via consensus. We manually coded the transcripts and also used Atlas.ti GmbH, Berlin version 7.5.10 (Student License) to manage the data [16]. Patient interviews were independently reviewed by the third author to ensure proposed recommendations accurately reflected patient needs.

## Results

### Participant characteristics

**Fig 3:**
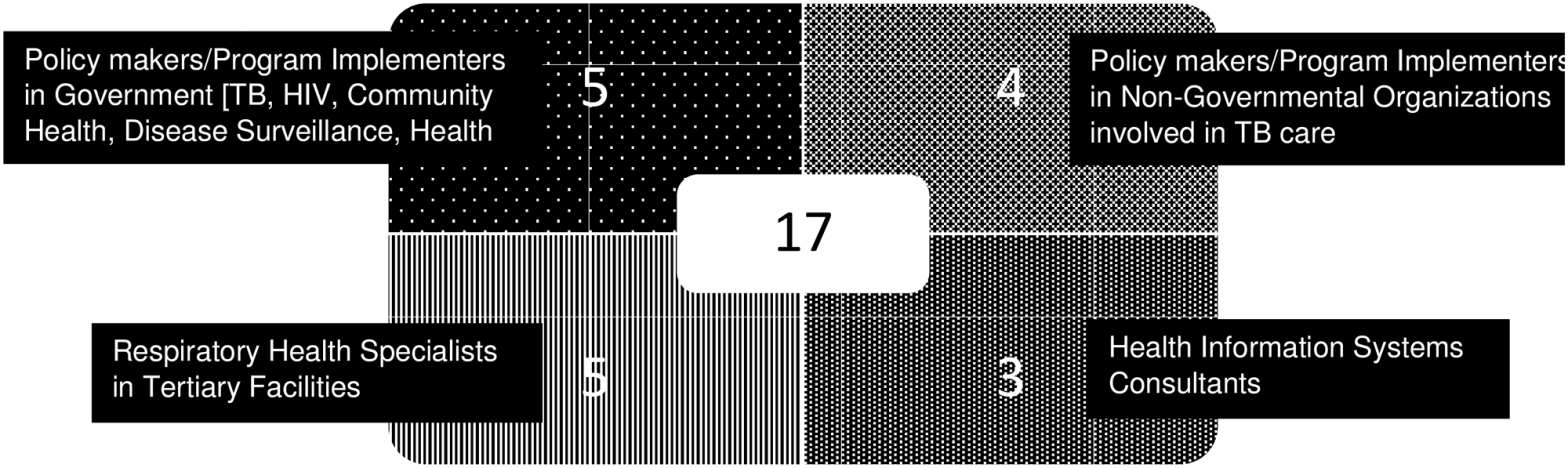
Key informant characteristics.

We conducted this qualitative study including 52 adult TB patients. As shown in Table 1, 27 (52%) were male, the median age was 33 years (IQR 26–40, range 21–50) and 36 (69%) were from primary health facilities. Thirty seven patients (71%) had attained eight complete years of primary school education and thirty five patients (67%) were employed/self-employed. Patients had diverse occupations including managers, administrators, marketers, shop assistants, hair stylists, tailors, mechanics, public vehicle drivers and conductors, barmaids, cleaners, casual laborers and businessmen/women selling cooked food, charcoal, khat and art among specified items. Three patients (6%) were college/university students. The remainder (27%) were unemployed comprising housewives, those with no jobs/looking for jobs and one refugee. Approximately two thirds of patients lived with either members of their nuclear or extended families. In 13 FGDs we conducted among HWs in each selected facility, the median number of participants was 7 HWs (IQR 6–8). (Table 2) Key informants involved in TB-CI related activities comprised 5 policy makers/implementers in government, 4 policy makers/implementers in non-governmental organizations, 5 respiratory health experts in pediatric and adult medicine including academic faculty in public and private sectors, and 3 health information system consultants. (Fig 3)

**Table 1:** TB patient characteristics.

Patient Characteristics	Total (n = 52)
Median age (IQR) [Range]	33 (26–40) [21,50]
Male (%)	27 (52)
Smear positive PTB (%)	44 (85)
Median duration in months since PTB diagnosis (IQR) [Range]	2.0 (1–3) [0.04,8]
Previously treated for TB and cured (%)	10 (19)
Second time TB diagnosis	8 (15)
Fourth time TB diagnosis	2 (4)
MDR TB	1 (2)
Level of education (%)	
No formal education	3 (6)
Incomplete primary education	12 (23)
Complete primary education	17 (33)
Complete secondary education	15 (29)
Complete tertiary education (polytechnic/college/university)	5 (10)
Occupation (%)	
Employed–upper tier [white collar] (manager, marketer, administrator, hotelier, messenger)	5 (10)
Employed–lower tier [blue collar] (hair stylist, tailor, shop assistant, mechanics, public vehicle drivers, public vehicle conductors, barmaids, cleaner, casual laborers)	20 (38)
Self-employed (businessmen/women: khat, charcoal, cooked food, tailor, art, unspecified)	10 (19)
Students (college/university)	3 (6)
Unemployed (housewives, no jobs/looking for jobs, refugee)	14 (27)
Primary health facility (%)	36 (69)
Sub-county (Health Facility Code)	
1. Dagoretti (I, II, V)	12 (4, 4, 4)
2. Embakasi (IX, XIII)	8 (4, 4)
3. Kamukunji (X)	4 (4)
4. Kasarani (XII)	4 (4)
5. Langata (III)	4 (4)
6. Makadara (VIII)	4 (4)
7. Njiru (VII)	4 (4)
8. Starehe (IV, XI)	8 (4, 4)
9. Westlands (VI)	4 (4)
Index TB patient-contact living dynamics (%)	
1. Live with their nuclear family in Nairobi	21 (40)
a. Husband/wife and children	17 (33)
b. Siblings	4 (8)
2. Live with their extended family in Nairobi	13 (25)
3. Live alone (single), has close contacts in Nairobi	10 (19)
a. Girlfriends/boyfriends	1 (2)
b. Neighbors	3 (9)
c. Workmates	3 (9)
d. College mates/friends	4 (8)
e. Relatives	4 (8)
4. Live alone (married), has family outside Nairobi	5 (10)
5. Travel out of Nairobi (frequently)	11 (21)
a. To visit husband/wife and children	5 (10)
b. To visit parents	5 (10)
6. Travel to Nairobi–to seek medical care	1 (2)

**Table 2:** Health worker focus group characteristics.

Health Worker Focus Group Characteristics	Total (N = 13 FGDs)													
Median Number of Participants (IQR) [Range]	7 (6–8) [4–10]													
Distribution of HWs per FGD	Total	I	II	III	IV	V	VI	VII	VIII	IX	X	XI	XII	XIII
Nurses	26	1	2	0	4	1	3	2	2	3	3	2	3	0
Clinical officers	15	1	1	0	1	1	1	1	0	2	1	2	1	3
HIV and psychosocial support counselors	15	3	1	0	1	1	0	4	1	2	1	0	1	0
Community health workers	11	0	0	1	0	1	1	1	2	2	1	0	1	1
Nutritionists	7	0	1	1	0	1	1	0	1	0	1	0	0	1
Lab technicians	6	0	0	1	1	1	0	1	0	0	1	1	0	0
Pharmacy technicians	2	0	0	1	0	0	0	1	0	0	0	0	0	0
Medical superintendent	1	0	0	0	0	0	0	0	0	0	0	0	0	1
Community health extension worker	1	0	0	0	0	0	0	0	0	0	0	1	0	0
Social worker	1	0	0	0	1	0	0	0	0	0	0	0	0	0
Total	85	5	5	4	8	6	6	10	6	9	8	6	6	6

### Index TB patients’ decision making on bringing contacts

From the individual patient interviews, most (37/52) patients seeking TB care at the facilities were invited by a HW to have their contacts screened and almost half (22/52) had at least one contact screened for TB. All TB patients whose contacts underwent screening were invited by a HW. Of 15 TB patients invited by a HW but did not have their contacts screened, 7 reported they would have their close contacts screened if they were symptomatic, 4 would bring at least one close contact on a specific date in the future, and the remainder reported: not feeling well /no finances to bring contacts/contacts busy. Approximately half of the TB patients who did not have their contacts screened but expressed willingness to come if invited. (Table 3)

**Table 3:** Patient identified facilitators and barriers to having contacts screened.

Reason	Number of Participants	Selected representative quote [participant code]
**Facilitators**		
Patient		
1. Understand TB	4	“For me, no (there are no barriers) because you know for infectious diseases, they are very dangerous. If you fail to take precautions, now the whole family will be destroyed. Me I don’t find any problem with that (my family being tested) because I love them and I need them. I want them to survive.” [P08]
2. Health benefits of contacts	4	“I live with my mother and this small child. Yes, here in Facility X, I was told to bring my mother and child to be checked for TB. I brought them for the benefit of their health.” [P32]
3. Contacts not to suffer	2	“I do not want my contacts to suffer like me.” [P051]
Community/Contacts		
1. Contact cares/support	1	“My brother came because he decided to, but also because he loves me and it’s also good that he came to learn more.” [P05]
2. Seen improved health	1	“Perhaps my mother saw the changes that happened to my health, and so she saw it was good she came. Like three weeks (of taking medicine is when she saw the changes)” [P32]
3. Treatment supporter	1	“Yes, there is a doctor who asked him (my brother) to be checked for TB, and he came. He came because I could have infected him, just in case. He is the one who brought me to hospital. So the doctor also told him to get tested.” [P19]
Heath System Service Delivery		
1. HW invitation	22	“I asked them (my family living in Mombasa) to go for testing for both HIV and TB. Yes, they (the doctors at the TB clinic) told me, in fact several times (to tell my family to get tested).” [P08]
2. Good service and kind	9	“They (health workers) handle me in a good way when I come here. They welcome me in a very nice way.” [P33]
3. Reverse contact investigation	2	“Yes, they found my TB when she (my daughter) came to the ward. So they (the doctors) sent me for the X-ray, it came out positive. I am a mother of three… Tomorrow before we get discharged the others have to come and get X-rays or tested if actually we have given it to them, and even if not, they is something they call anti-TB drugs which you have to take to prevent it.” [P30]
4. CHW home visits	1	“Yes, I was told to bring my brother for testing. The Community Health Worker explained to me. Sometimes they call us on phone, or they visit us at home. He explained to me that it is good they know where I live, they took our phone numbers so that in case they came and missed me, he would call me just to know how I’m fairing, and to visit us to know how the place we live in is like, and how the person we live with is like, many things about the home and living.” [P05]
5. Sputum container provision	1	“When I came, I was given a container for my husband to collect his sputum, and also for the children (in school, 10yrs and 8yrs). His (my husband’s) he removed (sputum) I returned it (to the facility) and he was found to be negative. [P06]
6. IPT provision	3	“I only have these two children. I don’t have a husband. Yes, the doctor told me to bring them so that they are tested if they have TB. This one is 9 years and this other one is two. They were tested in this facility. None of them has TB. The younger one was given medicine to prevent TB.” [P24]
**Barriers**		
Patients		
1. Feeling unwell	2	“I will bring them when I get better.” [P12]
2. Lack of money	2	“I will bring them when I get money.” [P37]
3. Lack of transport	2	“I don’t have transport”. [P16]
4. Busy at work	2	“I will bring them when I get an off from work.” [P09]
Community/Contacts		
1. Contact not agreeable	6	“For my husband, if he agrees, then he’ll come for a check-up.” [P41]; “The children I can take them. You know a woman is not a kid. If you like it, you can go, and if you don’t, you cannot force her.” [P23]
2. TB/HIV stigma	4	“You know sometimes some things you do secretly because people think that because you have TB you have HIV. Therefore, if you tell them, they will think you are positive so you try to be private.” [P04]
3. Busy contacts	2	“I don’t know when my husband will come for screening, because of work.” [P41]
4. Contact out of town	1	“My contacts are out of town.” [P43]
Health System Service Delivery		
1. Lack of HW invitation	15	“No one has ever asked me (to bring my close contacts for screening)” [P03]
2. Sub-optimal education	10	“No, I wasn’t explained about how TB is transmitted. No, I wasn’t given any health advice on TB. They just tested me for HIV. No, they didn’t tell me anything else.” [P10]
3. Sub-optimal enquiry	2	“No one has asked me if I have a family or not. If I was sent (to bring my close contacts for TB screening), because I understand that this (TB) is airborne, I would go and tell them that: ‘I have TB and it’s good that even you when you come you are tested because I don’t know if I could have given it to someone.’ I would explain it to them and they come. But the doctor hasn’t told me so I wouldn’t know.” [P03]
4. TB/HIV stigma	4	“They (the HWs) would tell you to sit outside. You know, they have a tent but you don’t sit inside the tent, you sit outside. You don’t expect someone to sit outside even if sunlight helps to kill the bacteria. You cannot keep me in the sun for four or five hours to kill that bacteria. And you know someone feels so bad! And they shout at you: ‘Go and sit there, go and sit there!’ It is not what you tell someone, it is how you tell them.” [P26]
5. Long wait-times	3	“There are long queues; the HWs are busy.” [P03]
6. Non-conducive clinic hours	2	“I’m not able to pick drugs over the weekend.” [P20]
7. Distant health facility	1	“This clinic is too far (my family is upcountry).” [P01]
8. No CHW home visit	1	“They (CHWs) never came (home). I think it was like last month [7th month of MDR treatment] he was still telling me, ‘Now we will be coming with that lady.’ So I was like, ‘You started telling me in October…November, December.’ So I told him, ‘I don’t think I’m really so important. You have taken so much time. [P26]

### Facilitators

**Fig 4:**
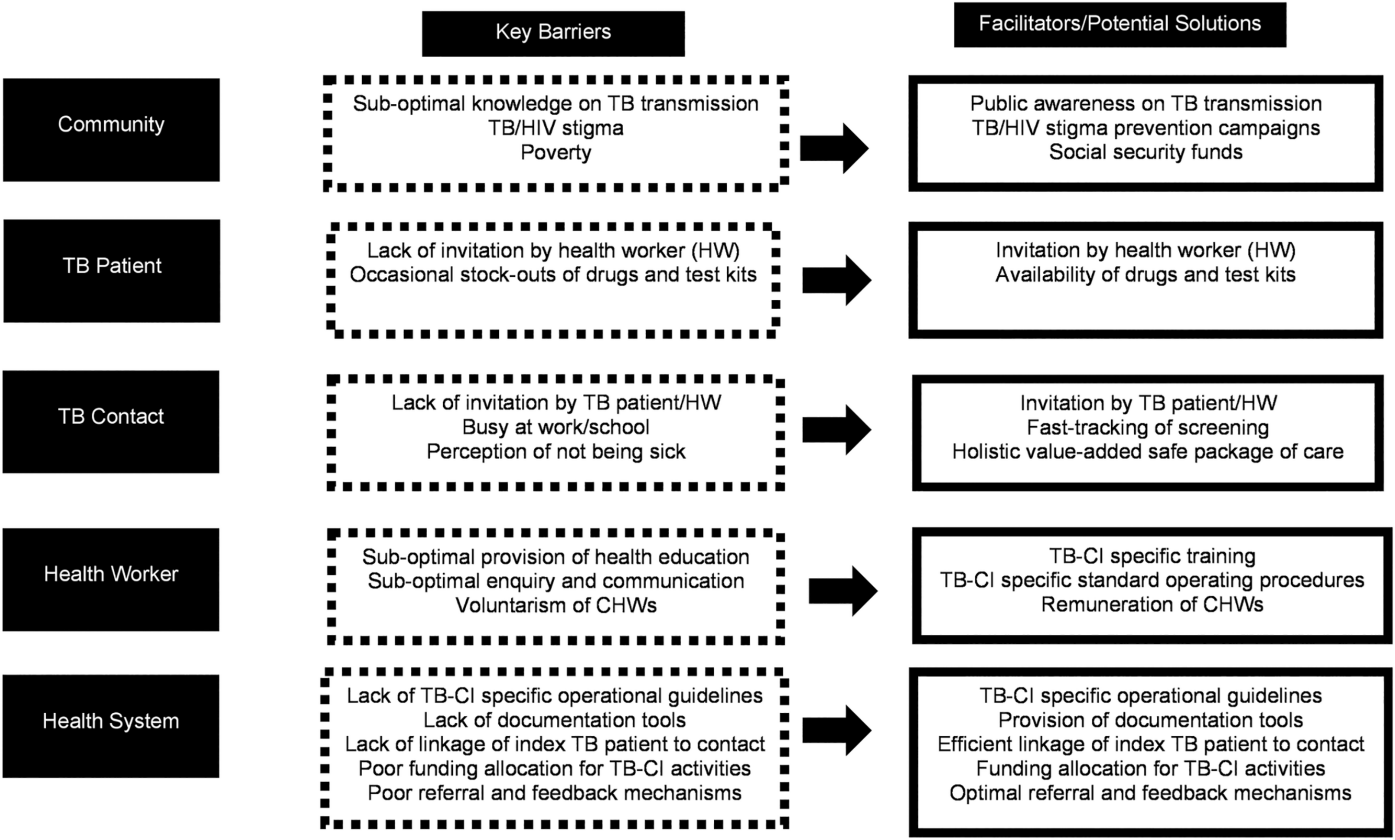
Operational framework for optimizing tuberculosis contact investigation.

Invitation of TB patients by HWs to bring close contacts was key to successful TB-CI. Patients reported that good rapport with HWs via flexibility, consideration, and generally handling patients well were facilitators for TB-CI. Additional facilitators included CHW home-visits and HW provision of sputum containers for unavailable contacts to overcome barriers such as contacts being at work or in school, and talking to family members, particularly husbands who were reported to be difficult to convince to come to the facility for TB-CI. Patients’ desire to avoid contacts suffering from TB, their concern for each other’s health, as well as the positive health changes that were seen once on treatment also promoted TB-CI activities. Screening of close contacts was also facilitated by the identification of TB in children (reverse CI). Furthermore, patients reported that HWs utilized TB-CI for provision of IPT for eligible contacts including children under the age of five years. Experts acknowledged strengths in the health system which included a resilient health workforce, supportive structures for service delivery, supervision in the TB, HIV and Community Health programs and strong multi-sectoral government leadership. (Tables 3 and 4; Fig 4)

**Table 4:** Facility observations, health worker and key informant perspectives on implementation of TB contact investigation.

WORLD HEALTH ORGANIZATION HEALTH SYSTEM BUILDING BLOCKS													
**1. Leadership and governance related**													
1a. Clear direction from the TB Program	Yes. *TB national guidelines call for invitation of close contacts of TB patients however operational guidelines offering a step by step approach for HWs, TB-CI specific documentation tools linking patients and their contacts, and a mechanism for patient and contact follow up are lacking.												
1b. Clear direction from the HIV Program	Yes. HIV national guidelines indicate that contacts of "open TB" patients should be screened, and under 5s regardless of HIV status given IPT if screen is negative; and all HIV patients should be screened for TB at each visit. Documentation for isoniazid prophylaxis is available.												
1c. Clear direction from the Community Health Program	Yes. *TB contact investigation in the community is undertaken if funded by partners/agencies. TB-CI specific documentation tools linking patients and their contacts and a mechanism for patient and contact follow up are available, however referral details are not provided to the patient for presentation and documentation at the health facility, relying on CHWs on physically accompanying TB contacts which although is regarded as the standard, is not feasible/hardly done.												
1d. Support from experts	Yes. *Experts in academia, public and private health sectors, non-governmental policy makers and policy implementers, and health system information consultants provide support. It was felt that government should lead with allocation of requisite resources particularly finances; and academia should be more actively involved in providing solutions. Experts propose provision of a holistic health care package that is flexible for close contacts of TB patients in different contexts.												
HEALTH FACILITY	**I**	**II**	**III**	**IV**	**V**	**VI**	**VII**	**VIII**	**IX**	**X**	**XI**	**XII**	**XIII**
1e. Support from health facility leaders	Yes	Yes	Yes	Yes	Yes	Yes	Yes	Yes	Yes	Yes	Yes	Yes	Yes
**2. Service delivery related**													
2a. Availability of operational guidelines for TB-CI	No	No	No	No	No	No	No	No	No	No	No	No	No
2b. TB patients agreeing to bring/inform contacts	Few	Few	Few	Some	Few	Some	Few	Few	Some	Few	Few	Some	Few
2c. Turnaround time optimal	No	No	Yes	Yes	Yes	Yes	No	No	No	Yes	Yes	Yes	Yes
2d. Integration of services (i. TB/HIV; ii. TB/Nutrition; iii. Other (e.g. psychosocial, rehabilitation, prison, FP)	i	i, ii	i, ii	i, ii, iii	i, ii	i, ii, iii	i, ii	i, ii	i, ii, iii	i, ii	i, ii	i, ii, iii	i, ii
2e. Focus on smear positive TB patients only	Yes	Yes	No	Yes	Yes	Yes	No	No	Yes	Yes	Yes	No	No
2f. Poor ventilation & lighting in TB waiting bays	No	No	Yes	No	Yes	No	Yes	No	No	No	No	Yes	Yes
**3. Supplies and products related**													
3a. Sputum containers	Yes	Yes	Yes	Yes	Yes	Yes	Yes	No^†^	No^†^	Yes	Yes	Yes	Yes
3b. Microscopy/Gene Xpert reagents	Yes^¥^	Yes^¥^	Yes	Yes	Yes	Yes	Yes	No^†^	No^†^	Yes	Yes	Yes	Yes
3c. HIV test kits	Yes	Yes	Yes	Yes	Yes*	Yes	Yes	Yes*	Yes*	Yes*	Yes*	Yes	Yes
HEALTH FACILITY (continued)	**I**	**II**	**III**	**IV**	**V**	**VI**	**VII**	**VIII**	**IX**	**X**	**XI**	**XII**	**XIII**
3d. Chest radiography	Yes	Yes	No	No	No	No	No	No	Yes	No	No	No	No
3e. TB drugs available	Yes	Yes	Yes*	Yes*	Yes*	Yes	Yes*	Yes*	Yes*	Yes*	Yes*	Yes	Yes
3f. Isoniazid prophylaxis: i. Infants; ii. Children; iii. HIV	i*	i*	ii, iii	ii, iii	ii, iii	iii*	iii*	ii, iii	ii, iii	iii*	ii, iii	ii, iii	ii, iii
3g. Nutrition therapeutic/supplementary feeds	No	Yes	Yes	Yes	Yes*	Yes*	Yes*	Yes*	Yes*	Yes*	Yes*	Yes	Yes*
**4. Health system financing related**													
4a.Presence of central budget support for TB-CI	No	No	No	No	No	No	No	No	No	No	No	No	No
4b. Partner support for TB-CI activities	No	No	Yes	Yes	Yes	Yes	Yes	Yes	Yes	Yes	Yes	No	Yes
**5. Health information system related**													
5a. Monitoring and supervision by NLTDP and NASCOP	Yes	Yes	Yes	Yes	Yes	Yes	Yes	Yes	Yes	Yes	Yes	Yes	Yes
5b. Standard report forms/registers for TB contact investigation provided by NTLDP and NASCOP	No	No	No	No	No	No	No	No	No	No	No	No	No
5c. TB contact investigation forms provided by an NGO	No	No	Yes	Yes	Yes	Yes	Yes	Yes	Yes*	Yes*	Yes*	No	Yes*
**6. Health workforce related**													
6a. Staff to monitor and evaluate TB-CI at national level	Yes	Yes	Yes	Yes	Yes	Yes	Yes	Yes	Yes	Yes	Yes	Yes	Yes
6b. Training of HCWs leading to confidence in TB-CI	No	No	Yes*	No	No	No	No	No	No	No	No	No	No
6c. Sufficient workload for HCWs	No	No	No	No	No	No	Yes	No	Yes	Yes	No	No	No
6d. Lack/inadequate CHW remuneration	N/A	Yes	Yes^α^	Yes^α^	Yes^α^	Yes^α^	Yes^α^	Yes^α^	Yes^α^	Yes^α^	Yes^α^	Yes	Yes
6e. Staff cohesion/team work & Passion for work done	Yes	Yes	Yes	Yes	Yes	Yes	Yes	Yes	Yes	Yes	Yes	Yes	Yes
PATIENT FACTORS													
**Barriers**													
Stigma (TB/HIV)–requiring community education	+++	+++	+++	+++	+++	+++^β^	+++	+++	+++	+++	+++	+++	+++
Poverty—difficulties accessing food, transport etc.	+++	+++	+++	+++	+++	+++	+++	-	+++	+++	+++	+	+++
Poor patient housing mainly slums with poor ventilation & lighting and overcrowding–requiring improvement	-	+++	+++	+++	+++	+++	+++	-	+++	+++	+++	-	+++
Drug addiction (including alcoholism) identified as an issue for TB patients	-	-	-	+++	-	+++	-	-	-	-	+++	-	-
**Solutions**													
Media for community education on TB-CI/reduce stigma	-	-	+++	+++	+++	-	+++	-	+++	+++	-	-	-
Proposed sustainable measures e.g. seed money for self-help groups/investment groups	-	-	-	+++	-	-	-	-	-	-	-	-	-
Incentives were an issue	+++^ø^	-	+++^§^	-	-	-	-	-	+++^ø^	+++^§^	-	-	+++**

^†^No lab;

^¥^No Gene Xpert reagents;

*Occasional stock-outs;

^α^Agency;

^β^Including stigma from HWs/staff;

^ø^Unsustainable;

^§^Promote stigma;

**Other patients found it unfair

### Barriers

Lack of operating procedures, documentation and HW training specific to TB-CI were the main barriers to its optimal implementation. On asking HWs whether TB patients brought their contacts/had their close contacts screened for TB, the response was either *“few”* or *“some”* in all the 13 FGDs. Sub-optimal HW enquiry of contacts noted from individual patient interviews, particularly those who lived alone, had child contacts who were asymptomatic/not part of the nuclear family, or close contacts out of town, may have led to lack of HW invitation. The TB register present in all TB clinics had a column for TB patients identified due to contact investigation, however this was rarely checked. There was no standardized tool in the facility for ascertaining that HWs had invited TB patients to have their contacts screened and neither was there a tool that linked TB patients to their contacts. During this study period, there were active TB-CI activities conducted in selected facilities in Nairobi County in the context of programs funded by non-governmental organizations and 10 of the 13 facilities were participating. Despite contacts in the community being referred to the facility for screening, they did not receive any documentation that would identify them to the facility as being referred for contact investigation, hence it was difficult to measure the efficacy of this active TB-CI activity. Similar to the passive TB-CI facility approach, a mechanism to link contacts to a particular TB patient was lacking, making it difficult to establish if all close contacts had been screened and estimate effectiveness of this strategy.

TB-related stigma among TB patients emerged as a major barrier to TB-CI and linkage to care in patient and expert interviews as well as all the HW FGDs (13/13). Patients generally had poor knowledge on the cause of TB and how it was transmitted. The presumption that all TB patients had HIV and misconceptions regarding TB transmission were common, including through sex, sharing utensils and genetics. CHWs cited experiencing near-violent encounters when visiting patients’ homes due to patients’ fear of their TB status being known by others, and others found that patients provide wrong home addresses and/or phone numbers to avoid CHWs visiting their homes–possibly fearing inadvertent disclosure. HWs in some facilities reported experiencing enacted stigma from colleagues who refused to work in the TB clinic areas for fear of infection. Few patients also reported HW enacted TB stigma while seeking TB care. Conversely, HWs reported they put themselves at risk for TB transmission by not putting on masks as they did not want to be seen to perpetuating stigma. (Tables 3–4; Fig 4)

### Opportunities for optimization

Key informants recognized the national TB program’s electronic surveillance and management system, TIBU, and proposed further innovation of the health management information system to support TB-CI that is user-friendly, efficient and interoperable. Experts explained that a national unique patient identifier was essential to avoid duplication of records and allow for linkage of data, and acknowledged that the government was taking the lead in this process. Additionally, stakeholders proposed fast-tracked care for contacts undergoing screening instituted in health facilities to avoid TB infection. Sustainable government-led funding for infrastructure, an adequate health workforce that is trained to deliver optimized TB-CI services, and social protection schemes to avert catastrophic costs were deemed imperative.

Considering program requirements and lessons learnt from stakeholder perspectives, specific areas of optimization that could be incorporated in facility based and/or community based TB CI strategies are outlined in Fig 4.

## Discussion

Through the lens of TB patients attending public TB clinics, HWs, key informants and facility observations, we were able to understand how TB-CI is undertaken in the capital city of a high TB/MDR/HIV burden country. Although CI has traditionally not been part of TB control efforts in LMICs [2], most TB patients in this context brought or encouraged their contacts to undergo TB screening if they were invited by a HW in both passive/active approaches. Similar to a study in Vietnam, approximately one-third of TB patients did not know about the need of investigating contacts [17]. Most of the TB patients not invited by HWs expressed willingness to bring their contacts if they had been invited, underscoring the fundamental role HWs play and a potential area for TB-CI optimization.

Despite HW invitation being key for screening of TB contacts, clear operational guidelines and procedures for systematic enquiry by HWs, and documentation tools were lacking. The minimum requirements for TB-CI information to be reported have been documented [3]. Standardizing protocols for TB-CI and training HWs to adhere to protocols has been shown to be useful in performing effective CIs and in conducting studies on the effectiveness of TB-CI [18, 19]. Moreover, the systematic enquiry in TB-CI as has been described in the literature as both a science and an art, requiring effective interviewing skills [18]. Based on findings from patient interviews we identified the following as important considerations during enquiry: HW kindness and confidentiality [20]; HWs employing tactful probes to establish close contacts; patient sociodemographic characteristics including living dynamics; patient knowledge on TB etiology, transmission, treatment and prevention [17]; and preventing TB-related stigma [21, 22]. Additionally, providing fast-tracked healthcare deemed valuable to caregivers/relatives/friends accompanying patients [23] and child contacts [24, 25] is imperative. Regardless of the TB-CI approach, infection control and prevention measures are required to prevent acquiring new infections [26].

We believe that our study provides a current contextual analysis of TB-CI activities, highlighting opportunities for optimization and may inform the design of interventions addressing barriers to TB-CI in similar contexts. We propose to utilize the framework developed from these study findings on a platform that allows for linkage of index TB patients to their contacts using unique patient identifiers to assess its utility in TB-CI activities.

## Conclusion

In conclusion, this study shows that TB contact invitation by HWs leading to contact screening does occur in the public health sector, however gaps exist. Key areas for optimization of TB-CI in this context include developing pragmatic TB-CI specific operational guidelines; a robust health information system that provides for documentation of TB-CI activities, linkage of index patients to their close contacts regardless of living dynamics, and referral and feedback loops; provision of holistic value added health packages for TB contacts undergoing TB screening, and sustainable government led community and health system interventions to promote TB-CI.

## Supporting information

S1 Table Observation flow mapping guide. (DOCX)

S2 Table Focus group discussion/interview guide. (DOCX)

S3 Table Consolidated criteria for reporting qualitative studies (COREQ). (DOCX)
