# Supplementary material for: Stakeholder perspectives for optimization of tuberculosis contact investigation in a high-burden setting
Source: PLoS One. 2017 Sep 8;12(9):e0183749. doi: 10.1371/journal.pone.0183749 (PMC5590832; doi:10.1371/journal.pone.0183749)
Supplement: S1 Table — (DOCX) [file pone.0183749.s001.docx]

**OBSERVATION & MAPPING GUIDE FOR TB CONTACT INVESTIGATION AND LINKAGE TO CARE**

| Date of form completion  **FACILITY:___________________________________**    **TIME PERIOD OBSERVED: _______________________**  dd mmm yyyy | |
| --- | --- |
| **TB CONTACT INVESTIGATION & LINKAGE TO CARE PROCESSES AND FLOW MAP** | **1. What methods are used?**  Facility based  Household based (CHWs)  Social network analysis  GPS/GIS  None  Other, specify__________  **2. Is there linkage to care?**  IPT  further TB testing  HIV testing  Nutrition care  Diabetes screening  Other, specify__________  **3. Barriers?**  **Patient:** stigma, clinic hours not conducive as at work, lack of transport/motivation, sub-optimal health education  **Health worker:** poor attitudes, knowledge and practices, documentation fatigue  **Health system:** lack of registers  Inability to link index to their contacts, patient delay (long patient times)  **4. Facilitators?**  **5. Solution opportunities?** |
| **6. What makes this method(s) work?**  Patient level  ________________________________________________________________________________________________________________________________________________________________________________________________________________________________________________________________________________________________________________________________________________________________________________________________________________________________________________________________________________________________  Health worker level  ________________________________________________________________________________________________________________________________________________________________________________________________________________________________________________________________________________________________________________________________________________________________________________________________________________________________________________________________________________________________  Health system level  ________________________________________________________________________________________________________________________________________________________________________________________________________________________________________________________________________________________________________________________________________________________________________________________________________________________________________________________________________________________________  **7. Are there any barriers to this method?**  Patient level  ________________________________________________________________________________________________________________________________________________________________________________________________________________________________________________________________________________________________________________________________________________________________________________________________________________________________________________________________________________________________  _______________________________________________________________________________________________________________________________________________________________________________________________  Health worker level  ________________________________________________________________________________________________________________________________________________________________________________________________________________________________________________________________________________________________________________________________________________________________________________________________________________________________________________________________________________________________  **________________________________________________________________________________________________________________________________________________________________________________________________**  Health system level  ________________________________________________________________________________________________________________________________________________________________________________________________________________________________________________________________________________________________________________________________________________________________________________________________________________________________________________________________________________________________  ________________________________________________________________________________________________  **8. Are there solution opportunities to these barriers? How would they work in this context at?**  **Patient Level**  Household visits  M-health  Non-monetary incentives: transport, food baskets  Monetary incentives  Health education  Other  _______________________________________________________________________________________________________________________________________________________________________________________________________________________________________________________________________________________________________________________________________________________________________________________________________________________________________________________________________________________________________________________________________________________________________________________  **Health worker level [specify cadre: __________________________ ]**  M-health  Non-monetary incentives: transport, food baskets  Monetary incentives  Better training on health education  Attitude change  Reduced number of registers  Reduced health worker risk  Other  ______________________________________________________________________________________________________________________________________________________________________________________________________________________________________________________________________________________________________________________________________________________________________________________________________________________________________________________________________________________________________________________________________________________________________________________________________  **Health system**  Reduced number of registers  Reduced health worker risk  Proper linkage of index patients/contacts to care – IPT, further TB testing, HIV testing, Nutrition & Diabetes screening  Proper linkage of data among health facilities  Shorter waiting times  Other  _____________________________________________________________________________________________________________________________________________________________________________________________________________________________________________________________________________________________________________________________________________________________________________________________________________________________________________________________________________________________________ | |
| **Filled in by ________(Initials) Date**  dd mmm yyyy | |

**WHO Health System Building Blocks**

*Patient, health worker and health system components that will be observed and explored include:-*

*i. Patient*

*1. Mapping TB patient and contact flow in the facility including linkage to care including IPT provision; further TB testing; HIV testing and care; nutrition screening and care; and diabetes screening and care.*

*2. Clinic hours and time taken at each process time.*

*ii. Health worker*

*1. Number of health workers involved in TB contact investigation and linkage to care.*

*2. Role health workers play in TB contact investigation and linkage to care in public areas in the health facility (observations not in settings individuals would reasonably expect privacy e.g. private consultation or examination).*

*3. General attitude of the health workers involved in TB contact investigation and linkage to care – confidence in care provision; propagation of stigma; work load.*

*4. TB patient file documentation of invitation of contacts for TB screening.*

*5. Filling of the TB contact register.*

*6. Documentation of TB patient linkage to care.*

*iii. Health system*

*1. Leadership and governance: policy support (posters or visual documentation related to TB contact investigation and linkage to care displayed at the facility).*

*2. Service delivery: availability of operational guidelines and details of TB contact investigation and linkage to care; availability of a TB contact register; ability of health worker to link the index TB patient to the contact [contact details e.g. phone number or physical address for both the index TB patient and the contact]; provision of IPT to eligible patients, further TB testing (chest radiography, mantoux test, sputum induction or gastric aspiration for children < 6 years/ patients who cannot produce a sputum sample), HIV testing, nutrition and diabetes screening.*

*3. Supplies and products: availability of mobile phone airtime; transport for health workers involved in TB contact investigation; IPT in the pharmacy including formulations available; reagents for TB testing in the lab [microscopy, GeneXpert], working chest x-ray machine and film availability, mantoux reagents, facility for sputum induction/gastric aspiration; Mid Upper Arm Circumference (MUAC) tapes, stadiometer, weighing scales, Kenyan Integrated Management of Acute Malnutrition (IMAM) screening charts for children and adults; functional glucometer and glucose strips.*

*4. Health system financing: Not able to observe this component.*

*5. Health information system: availability of a health information system (paper based, computerized or combination); availability of a mobile phone based system, global positioning system (GPS), geographic information system (GIS) or other system for TB contact investigation and linkage to care.*

*6. Health work force: (elaborated under health workers above); any visual documentation/posters on TB contact investigation and linkage to care trainings for health workers.*
