# Supplementary material for: Stakeholder perspectives for optimization of tuberculosis contact investigation in a high-burden setting
Source: PLoS One. 2017 Sep 8;12(9):e0183749. doi: 10.1371/journal.pone.0183749 (PMC5590832; doi:10.1371/journal.pone.0183749)
Supplement: S2 Table — (DOCX) [file pone.0183749.s002.docx]

**FOCUS GROUP DISCUSSION / INTERVIEW GUIDE**

| Date of form completion  **FACILITY/INSTITUTION:____________________________**    **PARTICIPANT CADRES: __________________________** dd mmm yyyy  TB patient TB contact Health worker (specify) Representative (specify) | | |
| --- | --- | --- |
| **TB CONTACT INVESTIGATION & LINKAGE TO CARE PROCESSES AND FLOW MAP**  Focus Group Discussion  * Introduction and ground rules.  * Ensure tape-recorder is on.  * Provide scenarios for TB contact investigation.  * Take questions, summarize discussion, thank participants and close.  * Highlight key process barriers and potential solutions below.  Interview  *Introductory patient script and hypothetical model attached.  Facility observations, Patient interviews, HW focus group discussions and Key informant interviews  *WHO health system building blocks attached. | | **SUMMARY**  **What methods are currently used?**  Facility based  Household based (CHWs)  Social network analysis  GPS/GIS  None  Other, specify__________  **Is there linkage to care?**  IPT  further TB testing  HIV testing  Nutrition care  Diabetes screening  None  Other, specify__________  ***If highlighted barriers are brought up explore deeper**  **BARRIERS?**  **Patient:** stigma, clinic hours not conducive as at work, lack of transport/motivation, sub-optimal health education  **Health worker:** irregular trainings/ guideline dissemination, documentation fatigue, poor attitude  **Health system:** lack of registers  Inability to link index to their contacts, patient delay (long patient times)  **FACILITATORS/ SOLUTIONS?**  **Patient:** household visits, incentives, m-health, peer support, local entrepreneurs  **Health worker:** incentives, m-health, increased staffing, regular staff training, timely guideline dissemination  **Health system:** linked registers, unnecessary processes eliminated, better linkage of care, GIS, GPS, social networks, data privacy |
| EXPLORING FACILITATORS, BARRIERS AND POTENTIAL SOLUTIONS TO TB CONTACT INVESTIGATION  AND LINKAGE TO CARE IN NAIROBI, KENYA | |  |
| **1. What type of TB contact investigation methods are used in your facility?**  Facility based Household based Social network analysis GPS/GIS None  Other, specify ____________________________________________    **2. Is there linkage to care?**  IPT Further TB testing HIV testing Nutrition screening Diabetes screening None  Other, specify ____________________________________________  **3. What makes this method(s) work?**  Patient level  ___________________________________________________________________________________________________________________________________________________________________________________________________________________________________________________________________________________________________________________________________________________________________________________________________________________________________________________________________________________________  Health worker level  __________________________________________________________________________________________________________________________________________________________________________________________________________________________________________________________________________________________________________________________________________________________________________________________________________________________________________________________________________________________  Health system level  __________________________________________________________________________________________________________________________________________________________________________________________________________________________________________________________________________________________________________________________________________________________________________________________________________________________________________________________________________________________  **4. Are there any barriers to this method?**  Patient level  __________________________________________________________________________________________________________________________________________________________________________________________________________________________________________________________________________________________________________________________________________________________________________________________________________________________________________________________________________________________________________________________________________________________________________________________  _____________________________________________________________________________________________________________________________________________________________________________________________________________________________________________________________________________________________  Health worker level  __________________________________________________________________________________________________________________________________________________________________________________________________________________________________________________________________________________________________________________________________________________________________________________________________________________________________________________________________________________________________________________________________________________________________________________________  **_____________________________________________________________________________________________________________________________________________________________________________________________________________________________________________________________________________________________**  Health system level  __________________________________________________________________________________________________________________________________________________________________________________________________________________________________________________________________________________________________________________________________________________________________________________________________________________________________________________________________________________________________________________________________________________________________________________________  _____________________________________________________________________________________________________________________________________________________________________________________________  **5. Are there potential solutions to these barriers? How would they work in this context?**  **Patient Level**  Household visits  M-health  Non-monetary incentives: transport, food baskets, other (specify)_________________  Monetary incentives  Health education  Other  ___________________________________________________________________________________________________________________________________________________________________________________________________________________________________________________________________________________________________________________________________________________________________________________________________________________________________________________________________________________________  **Health worker level [specify cadre: __________________________ ]**  M-health  Non-monetary incentives: transport, other (specify)_________________  Monetary incentives  Frequent re-fresher training on health education  Timely guideline dissemination  Attitude change  Reduced number of registers/ Efficient or linked registers  Reduced health worker risk  Other  ________________________________________________________________________________________________________________________________________________________________________________________________________________________________________________________________________________________________________________________________________________________________________________________________________________________________________________________________________________________________________________________________________________________________________________________________________________________________________________________________________________________ | |  |

| **Health system**  Reduced number of registers  Reduced health worker risk  Proper linkage of index patients/contacts to care – IPT, further TB testing, HIV testing, Nutrition & Diabetes screening  Proper linkage of data among health facilities  Computerized registers  Shorter waiting times  Other  _______________________________________________________________________________________________________________________________________________________________________________________________________________________________________________________________________________________________________________________________________________________________________________________________________________________________________________________________________________________________________________________________________________________________________________________________________________________________________________________________________________________________________________________________________________________________________________________________  **6. Social Network Analysis [Explain what this means]**   \| **ADVANTAGES** \| **BARRIERS** \| **POTENTIAL SOLUTIONS** \| \| --- \| --- \| --- \| \|  \|  \|  \| \|  \|  \|  \| \|  \|  \|  \| \|  \|  \|  \| \|  \|  \|  \| \|  \|  \|  \| \|  \|  \|  \| \|  \|  \|  \| \|  \|  \|  \| |
| --- | --- | --- | --- | --- | --- | --- | --- | --- | --- | --- | --- | --- | --- | --- | --- | --- | --- | --- | --- | --- | --- | --- | --- | --- | --- | --- | --- | --- | --- | --- |

| **7. Global positioning systems and Geographical information systems [Explain what this means]**   \| **ADVANTAGES** \| **BARRIERS** \| **POTENTIAL SOLUTIONS** \| \| --- \| --- \| --- \| \|  \|  \|  \| \|  \|  \|  \| \|  \|  \|  \| \|  \|  \|  \| \|  \|  \|  \| \|  \|  \|  \| \|  \|  \|  \| \|  \|  \|  \| \|  \|  \|  \|   **8. Would you like to share anything else regarding our discussion on TB contact investigation and linkage to care?**  _______________________________________________________________________________________________________________________________________________________________________________________________________________________________________________________________________________________________________________________________________________________________________________________________  *________________________________________________________________________________________________*  *Closure- thank the participant for their time* |
| --- | --- | --- | --- | --- | --- | --- | --- | --- | --- | --- | --- | --- | --- | --- | --- | --- | --- | --- | --- | --- | --- | --- | --- | --- | --- | --- | --- | --- | --- | --- |
| **Interview conducted by ________(Initials) Date**  dd mmm yyyy |

**Introductory Patient Interview Script and Hypothetical Decision Model**

| Introduction and establishing rapport | Hello, My name is _________*.*  Which language do you prefer for us to talk in? [Probe: English, Swahili, Other (specify)] |
| --- | --- |
| Diagnosis of PTB | *[Verify with patient file or TB card]* Sputum positive? MDR TB? Previously treated for TB? |
| Social demographic characteristics | How old are you? *When were you born? [Probe: Date of birth]  What is the highest level of education you have attained?  What do you do for a living? [Probe: What work do you do? Do you go to school?]  Are you married or in an intimate relationship? Do you have children?  Where do you live? What’s the nearest public health facility to you? |
| Current living dynamics and contact characteristics | How many people live in your house currently? Who do you live with? [Probe]:-  What is your relationship? How old is he/she? Does he/she currently live in Nairobi? What work does she/he do?  Does your spouse/boyfriend/girlfriend live with you?  Are there any children who live with you? Who takes care of them? Do you have a Nanny/House-help?  Do you live with your relatives?  Do you travel to visit your family members? [Specify: Nuclear family? Other relatives?]  How often do you travel to visit your family members in a month? How long do you spend with them in a month? |
| Assessing other current close contacts beyond the household | Where do you spend most of your time?  [Probe: At home? With friends? With neighbours? At work? At school? (In prison?) Other, specify?]  Are there other people you have lived with (apart from those mentioned above)?  Are there other people you have been in close contact with (apart from those mentioned above)?  Are there any children who have been in close contact with (apart from those mentioned above)?  Is there any of your contacts who are sick? |
| TB diagnosis | When did you find out that you had TB? (Which date? In which facility were you diagnosed? Tell me what happened…. [then refer to decision model] |
|  | |

**WHO Health System Building Blocks**

*Patient, health worker and health system components that will be observed and explored include:-*

*i. Patient*

*1. Mapping TB patient and contact flow in the facility including linkage to care including IPT provision; further TB testing; HIV testing and care; nutrition screening and care; and diabetes screening and care.*

*2. Clinic hours and time taken at each process time.*

*ii. Health worker*

*1. Number of health workers involved in TB contact investigation and linkage to care.*

*2. Role health workers play in TB contact investigation and linkage to care in public areas in the health facility (observations not in settings individuals would reasonably expect privacy e.g. private consultation or examination).*

*3. General attitude of the health workers involved in TB contact investigation and linkage to care – confidence in care provision; propagation of stigma; work load.*

*4. TB patient file documentation of invitation of contacts for TB screening.*

*5. Filling of the TB contact register.*

*6. Documentation of TB patient linkage to care.*

*iii. Health system*

*1. Leadership and governance: policy support (posters or visual documentation related to TB contact investigation and linkage to care displayed at the facility).*

*2. Service delivery: availability of operational guidelines and details of TB contact investigation and linkage to care; availability of a TB contact register; ability of health worker to link the index TB patient to the contact [contact details e.g. phone number or physical address for both the index TB patient and the contact]; provision of IPT to eligible patients, further TB testing (chest radiography, mantoux test, sputum induction or gastric aspiration for children < 6 years/ patients who cannot produce a sputum sample), HIV testing, nutrition and diabetes screening.*

*3. Supplies and products: availability of mobile phone airtime; transport for health workers involved in TB contact investigation; IPT in the pharmacy including formulations available; reagents for TB testing in the lab [microscopy, GeneXpert], working chest x-ray machine and film availability, mantoux reagents, facility for sputum induction/gastric aspiration; Mid Upper Arm Circumference (MUAC) tapes, stadiometer, weighing scales, Kenyan Integrated Management of Acute Malnutrition (IMAM) screening charts for children and adults; functional glucometer and glucose strips.*

*4. Health system financing: Not able to observe this component.*

*5. Health information system: availability of a health information system (paper based, computerized or combination); availability of a mobile phone based system, global positioning system (GPS), geographic information system (GIS) or other system for TB contact investigation and linkage to care.*

*6. Health work force: (elaborated under health workers above); any visual documentation/posters on TB contact investigation and linkage to care trainings for health workers.*
